# T-cell redirecting bispecific antibodies targeting BCMA for the treatment of multiple myeloma

**DOI:** 10.18632/oncotarget.27792

**Published:** 2020-11-10

**Authors:** Christie P.M. Verkleij, Kristine A. Frerichs, Marloes Broekmans, Saida Absalah, Patricia W.C. Maas-Bosman, Sandy Kruyswijk, Inger S. Nijhof, Tuna Mutis, Sonja Zweegman, Niels W.C.J. van de Donk

**Affiliations:** ^1^Amsterdam UMC, Vrije Universiteit Amsterdam, Department of Hematology, Amsterdam, The Netherlands; ^*^Shared first authors

**Keywords:** multiple myeloma, bispecific antibody, immunotherapy, BCMA, CD38

## Abstract

B-cell maturation antigen (BCMA)-targeting bispecific antibodies and bispecific T-cell engagers (BiTEs) redirect T-cells to BCMA-expressing multiple myeloma (MM) cells. These MM cells are subsequently eliminated via various mechanisms of action including the release of granzymes and perforins. Several phase 1, dose-escalation studies show pronounced activity of BCMA-targeting bispecific antibodies, including teclistamab, AMG420 and CC-93269, in heavily pretreated MM patients. Cytokine release syndrome is the most common adverse event, which can be adequately managed with tocilizumab or steroids. Several clinical trials are currently evaluating combination therapy with a BCMA-specific bispecific antibody, based on preclinical findings showing that immunomodulatory drugs or CD38-targeting antibodies enhance the activity of bispecific antibodies. In addition, bispecific antibodies, targeting other MM cell surface antigens (i. e. GPRC5D, CD38 and FcRH5), are also evaluated in early phase clinical trials. Such bispecific antibodies, targeting other antigens, may be given to patients with low baseline BCMA expression, disease with substantial heterogeneity in BCMA expression, following prior BCMA-targeted therapy, or combined with BCMA bispecific antibodies to prevent development of antigen escape.

## INTRODUCTION

### Immunotherapy in multiple myeloma

**Figure 1:**
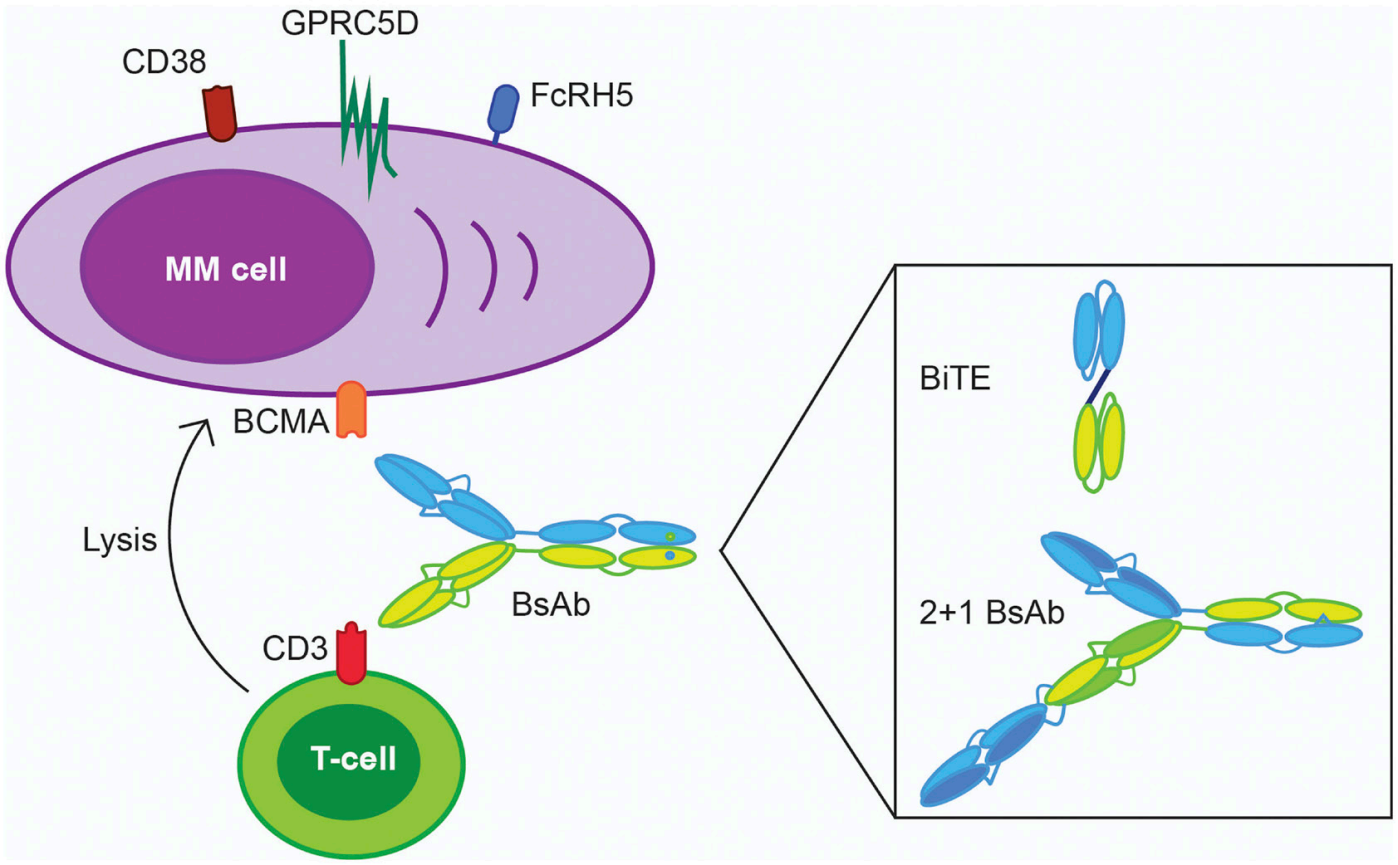
Examples of bispecific antibody designs used in clinical trials with multiple myeloma patients. Bispecific antibodies (BsAbs) bind with one arm to a CD3 antigen expressed on the T-cell surface and with another arm to a tumor-associated antigen (TAA) expressed on MM cells, such as B-cell maturation antigen (BCMA), CD38, G protein-coupled receptor of family C, group 5, member D (GPRC5D), and Fc receptor-homolog 5 (FcRH5). This coupling results in activation and degranualtion of T-cells and subsequent lysis of MM cells. Alternatives to the “classic” bivalent IgG-like BsAb design (e.g., teclistamab) are bispecific T-cell engagers (BiTEs, e.g., AMG420), which are comprised of two single-chain fragment variables and a peptide linker, and lack an Fc domain; and a 2+1 BsAb design (e.g., CC-93269), which is characterized by one bivalent arm that binds to both CD3 and a TAA and a monovalent arm that connects with a TAA.

Immunotherapeutic agents are increasingly used for the treatment of both newly diagnosed and relapsed/refractory multiple myeloma (MM) patients [1–3]. This includes regimens containing CD38-targeting antibodies (e.g., daratumumab or isatuximab) for the treatment of newly diagnosed, transplant-eligible and transplant-ineligible, MM patients [4–6]. Furthermore, combination therapy with a CD38-targeting antibody or a SLAMF7-targeting antibody (elotuzumab) can also be used for patients with relapsed/refractory MM [7–11]. In addition, the FDA and EMA recently approved the B-cell maturation antigen (BCMA)-targeting antibody-drug conjugate, belantamab mafodotin, for the treatment of patients with advanced, heavily pretreated, MM [12]. Although these different immunotherapies have contributed to an improved outcome of MM patients, those with disease refractory to immunomodulatory drugs (IMiDs), proteasome inhibitors (PIs) and CD38-targeting antibodies have a very poor survival of less than one year [13]. The worst prognosis is observed in penta-refractory patients (refractory to 2 IMiDs, 2 PIs, and a CD38-targeting antibody) with a median overall survival of only 5.6 months. Importantly, T-cell redirecting therapies, such as chimeric antigen receptor (CAR) T-cells and bispecific antibodies (Figure 1), have significant clinical activity in these heavily pretreated patients.

### Preclinical data on BCMA-targeting bispecific antibodies and BiTEs

We have recently provided the preclinical rationale for the use of the BCMAxCD3 bispecific antibody teclistamab (JNJ-7957) [14]. Teclistamab activates T-cells by connecting the CD3 antigen, present on T-cells, with BCMA, which is highly and selectively expressed on the surface of normal and clonal plasma cells. The redirected T-cells kill MM cells via various mechanisms including the release of perforins and granzymes. Teclistamab-mediated T-cell activation also resulted in a dose-dependent increase in several cytokines such as interferon-γ, tumor necrosis factor-α, interleukin-6, and interleukin-2. We also demonstrated that teclistamab effectively killed primary MM cells. Importantly, lysis was not only observed in samples obtained from newly diagnosed MM patients, but also in samples from heavily pretreated patients. This is a relevant finding since T-cell function in heavily pretreated patients is impaired due to the cumulative exposure to immunosuppressive anti-MM drugs (i.e., dexamethasone, proteasome inhibitors and alkylating drugs). In addition, patients with advanced disease have a more immunosuppressive tumor microenvironment with increased regulatory T-cell counts, as well as upregulation of inhibitory immune checkpoints, such as PD-L1. Because teclistamab-mediated MM cell lysis was heterogeneous, we assessed the impact of various tumor- and host-related factors on *ex vivo* response. The efficacy of teclistamab was not associated with cell surface expression of BCMA or cytogenetic risk status. However, samples with a high number of T-cells and thereby a higher T-cell to tumor cell ratio had superior teclistamab-mediated lysis, when compared to samples with low numbers of T-cells or low effector: target ratio. In agreement with our results, other bispecific antibodies or bispecific T-cell engagers (BiTE) targeting BCMA also showed high anti-MM activity in preclinical models [15–17].

### Clinical studies with BCMA-targeting bispecific antibodies and BiTEs

Teclistamab is currently evaluated in a first-in-man study in heavily pretreated MM. The first results show high single agent activity and a favorable toxicity profile [18]. Teclistamab was administered via intravenous infusion to 78 patients with a median of 6 prior lines of therapy (80% of patients was triple-class refractory). Response was dose-dependent, with no responses observed in patients receiving teclistamab at a dose of 19.2 μg/kg or less, while the overall response rate was 30% in patients treated with 38.4–180 μg/kg. The best response was reported for the 12 patients treated at the dose of 270 μg/kg (highest dose reported up till now): at least partial response (PR) in 67%, at least very good partial response in 50%, and complete response (CR) in 25%. The most common adverse event related to teclistamab was cytokine release syndrome (CRS), which occurred in 56% of patients. Importantly, no grade ≥ 3 CRS was observed in these patients and most CRS events occurred during the step up dosing or first full dose [18]. Other toxicities included cytopenias and infections. The study is ongoing and currently exploring higher doses of teclistamab, as well as a subcutaneous administration, which may be the preferred route of administration because it is less invasive and faster, and will thereby reduce burden placed on the day care unit.

Preliminary data from other clinical trials also shows promising activity of BCMA-targeting bispecific antibodies and BiTEs [15]. One of these bispecific antibodies is CC-93269, which is characterized by bivalent binding to BCMA. The most recent update of the phase 1 study with this drug, reported results from the first 30 patients [19]. These patients had received a median of 5 prior lines of therapy (67% triple-class refractory). Similar to teclistamab, response to CC-93269 was dose-dependent with an overall response rate of 43% in all patients and 89% in the 9 patients treated with 10 mg CC-93269 including CR in 44%. CRS was observed in 77% of the patients, including one grade ≥ 3 event. Other adverse events included infections and cytopenias. This trial is ongoing to define the recommended phase 2 dose level.

AMG420 is the first-in-class BCMA-targeting BiTE. AMG420 has a short half-life because of its small size, and therefore needs to be administered via continuous intravenous infusion during 4 weeks of each 6-week cycle [20]. In the dose-escalation trial with 42 relapsed/refractory MM patients (median of 3.5 prior lines of therapy; 21% daratumumab-refractory), AMG420 induced a high response rate (≥ PR at the maximum-tolerated dose of 400 μg/day: 70%). CRS occurred in 38% of patients. Other adverse events included infections, bone marrow suppression, and polyneuropathy. Clinical development of AMG420 is stopped, because continuous intravenous infusion is logistically challenging. A half-life extended BCMA-targeting BiTE molecule, AMG701, is currently being evaluated in an ongoing phase 1 trial.

## FUTURE DEVELOPMENTS

### Combination treatment

Combination therapy is often used in cancer to prevent the outgrowth of resistant clones. Because of the high single agent activity and good tolerability profile of the BCMA-targeting bispecific antibodies, these agents are ideal components of a highly effective combination therapy. We showed that the direct combination of teclistamab with the CD38-targeting antibody daratumumab enhanced MM cell lysis in an additive fashion [14]. In addition, the activity of teclistamab was significantly enhanced in bone marrow samples obtained from patients with prior daratumumab treatment. Furthermore, teclistamab-mediated tumor cell lysis was superior when T-cells obtained from patients treated with daratumumab were used, compared to T-cells from daratumumab naïve MM patients [14]. This positive effect of daratumumab on the ability of teclistamab to kill MM cells, is probably related to the immune stimulating effects of daratumumab, which include the elimination of immune suppressor cells, induction of T-cell expansion, and increase in T-cell killing capacity [14, 21–23]. These data form the rationale for the ongoing phase 1 TRIMM study, which evaluates the efficacy of teclistamab plus daratumumab.

IMiDs, such as lenalidomide and pomalidomide, also have T-cell stimulating effects [24]. Indeed, pretreatment of effector cells with lenalidomide or pomalidomide enhanced AMG701-mediated lysis of MM cells [25, 26]. These data provide the rationale for clinical trials evaluating the combination of a bispecific antibody or BiTE with an IMiD. The cereblon E3 ligase modulator iberdomide (CC220) is a more potent immune stimulatory drug, compared to lenalidomide and pomalidomide [27], and is therefore also a promising potential combination partner for bispecific antibodies.

### Earlier lines of therapy

Because of the high activity and favorable toxicity profile of bispecific antibodies in end-stage MM, these agents may also be beneficial for patients with early relapsed/refractory MM. Furthermore, newly diagnosed MM patients with high-risk cytogenetic abnormalities, who have a poor prognosis, may benefit from the incorporation of a BCMA-targeting bispecific antibody into their upfront treatment strategy. This is also supported by our finding that the *ex vivo* activity of teclistamab was not affected by presence of high-risk cytogenetic abnormalities. In addition, newly diagnosed patients, who do not achieve a deep response with a standard-of care treatment approach, may benefit from early access to a BCMA-targeting bispecific antibody with as ultimate goal the achievement of minimal residual disease-negativity.

### Other bispecific and trispecific antibodies

Although the BCMA-targeting bispecific antibodies are most advanced in terms of clinical testing, several other T-cell redirecting antibodies are evaluated in early clinical trials. This includes bispecific or trispecific antibodies targeting GPRC5D, CD38, and FcRH5 [28–33].

Bispecific antibodies targeting other MM-associated antigens may be useful in patients with low baseline BCMA expression or heterogeneous BCMA expression. In these patients treatment with a BCMA-targeting bispecific antibody may lead to rapid outgrowth of BCMA^low^ clones. Furthermore, although a case report suggests that serial treatment with BCMA-targeting agents is feasible [34], we need more information on this important issue. If prior treatment with BCMA-targeted CAR T-cells or a BCMA-targeting antibody drug conjugate contributes to resistance to a BCMA-targeting bispecific antibody via reduced BCMA cell surface expression, then treatment with a bispecific antibody targeting another antigen may be more effective. In addition, a combination of bispecific antibodies, simultaneously targeting different antigens, may mitigate tumor antigen escape.

## CONCLUSIONS

BCMA-targeting bispecific antibodies are highly active in extensively pretreated patients, including those who are refractory to all available drugs. These agents have a favorable safety profile, with CRS as most common adverse event, which can be easily managed with tocilizumab or steroids. A major advantage of bispecific antibodies or BiTEs over CAR T-cells is their direct “off-the-shelf” availability, while production of CAR T-cells takes several weeks. However, treatment with bispecific antibodies is typically continued until progression, while CAR T-cells are generally given as a single infusion and consequently patients will have a treatment-free interval, which is often preferred by patients and care givers. Finally, bispecific antibodies are promising combination partners with other immune stimulating drugs such as IMiDs and CD38-targeting antibodies. Clinical trials evaluating these combinations are ongoing.
